# Information theoretic measures for quantifying sequence–ensemble relationships of intrinsically disordered proteins

**DOI:** 10.1093/protein/gzz014

**Published:** 2019-08-03

**Authors:** Megan C Cohan, Kiersten M Ruff, Rohit V Pappu

**Affiliations:** Department of Biomedical Engineering and Center for Science & Engineering of Living Systems (CSELS) Washington University in St. Louis, One Brookings Drive, Campus Box 1097, St. Louis MO, USA

**Keywords:** computations, ensemble entropy matrix, intrinsically disordered proteins, protein design, sequence-ensemble relationships

## Abstract

Intrinsically disordered proteins (IDPs) contribute to a multitude of functions. *De novo* design of IDPs should open the door to modulating functions and phenotypes controlled by these systems. Recent design efforts have focused on compositional biases and specific sequence patterns as the design features. Analysis of the impact of these designs on sequence-function relationships indicates that individual sequence/compositional parameters are insufficient for describing sequence-function relationships in IDPs. To remedy this problem, we have developed information theoretic measures for sequence–ensemble relationships (SERs) of IDPs. These measures rely on prior availability of statistically robust conformational ensembles derived from all atom simulations. We show that the measures we have developed are useful for comparing sequence-ensemble relationships even when sequence is poorly conserved. Based on our results, we propose that *de novo* designs of IDPs, guided by knowledge of their SERs, should provide improved insights into their sequence–ensemble–function relationships.

## Introduction

Advances in *de novo* design (Baker, 2019) have given rise to proteins with new folds (Pessi *et al.*, 1993; Smith and Hecht, 2011; ElGamacy *et al.*, 2018), novel functions (Khare and Fleishman, 2013), controllable dynamics (Johansson and Lindorff-Larsen, 2018; Kundert and Kortemme, 2019) and unnaturally high stabilities (Geiger-Schuller *et al.*, 2018; Marcos *et al.*, 2018). Historically, protein design was cast as an inverse protein-folding problem (Ponder and Richards, 1987; Bowie *et al.*, 1991; Yue and Dill, 1992; Kuhlman and Baker, 2004), whereby one prescribes a structure or a fold and uses design principles to uncover the family of sequences that are compatible with the fold. In this approach, one seeks the set of sequences for which the free energy of folding, defined as the difference between standard state free energies of the folded and unfolded states, is negative. In addition to being able to design sequences that are compatible with a prescribed fold, modern tools in protein design are yielding novel folds with bespoke functions leading to a revolution in synthetic biology (Pleiss, 2011; Gainza-Cirauqui and Correia, 2018).

Advances in protein design may be traced to improvements in our understanding of sequence–structure–function relationships of proteins (Shakhnovich *et al.*, 2003; Qi and Grishin, 2005; Fischer *et al.*, 2011; Marchler-Bauer *et al.*, 2011). These improvements are manifest in being able to codify relationships between sequence and structure. Improvements in *de novo* structure prediction, which essentially represent the ability to relate sequence to structure, have also gone a long way toward enabling rapid advances in protein design (Heinke *et al.*, 2013). When fluctuations around well-ordered structures have to be incorporated into the designs, one can enhance computational design strategies using novel Monte Carlo sampling (Mignon and Simonson, 2016; Kundert and Kortemme, 2019).

The preceding narrative summarizes the state-of-the-art for the design of proteins that spontaneously adopt well-defined folds under typical folding conditions. We refer to these proteins as intrinsically ordered proteins (IOPs). However, over a third of eukaryotic proteomes feature proteins or regions that are defined by significant conformational heterogeneity and are referred to as intrinsically disordered proteins/regions (IDPs/IDRs) (Wright and Dyson, 1999, 2015; Dunker *et al.*, 2002; Forman-Kay and Mittag, 2013; van der Lee *et al.*, 2014). It is becoming increasingly clear that IDPs/IDRs have important functional roles, especially in the context of controlling the outcomes of decision-making and influencing circuits in cells. Therefore, it stands to reason that IDPs/IDRs provide a prime target for protein design. However, it is often the case that sequences of IDPs/IDRs are poorly conserved, even when they belong to the same functional family across orthologs (Brown *et al.*, 2011; Moesa *et al.*, 2012; Lange *et al.*, 2016). This stands in direct contrast to IOPs, where proteins that contribute to similar functions often have similar sequences (Pirovano and Heringa, 2008) and hence similar structures, although numerous exceptions to this rule do exist (Bryan and Orban, 2010; Wasserman and Saphire, 2016). Despite these exceptions, multiple sequence alignments (MSAs) are highly informative for inferring sequence-structure relationships of IOPs, but they are not very useful in classifying IDPs/IDRs unless considerable prior knowledge is brought to bear on designing substitution matrices that are used in sequence alignments (Lange *et al.*, 2016).

It has been observed, however, that IDPs/IDRs retain similarities in terms of amino acid compositions, even when sequence similarities are minimal (Brown *et al.*, 2011; Moesa *et al.*, 2012). This has lead to the development and deployment of various tools that enable the computation of compositional parameters for IDPs/IDRs (Holehouse *et al.*, 2017). These include parameters such as average hydropathy, the fraction of charged residues (FCR), the net charge per residue (NCPR) (Das *et al.*, 2015), the patterning of oppositely charged residues along the linear sequence (Das and Pappu, 2013; Sawle and Ghosh, 2015; Firman and Ghosh, 2018), and the patterning of proline and charged residues vis-à-vis other residues (Martin *et al.*, 2016).

Sequences of IDPs/IDRs can be compared to one another in terms of coarse-grained compositional parameters or by comparing compositional profiles written out along sliding windows (Holehouse *et al.*, 2017). Recent efforts have focused on *de novo* redesigns of specific IDRs by altering compositional biases and patterning parameters to influence overall dimensions, specific molecular functions, phase behavior, and cellular phenotypes (Beh *et al.*, 2012; Nott *et al.*, 2015; Das *et al.*, 2016; Martin *et al.*, 2016; Pak *et al.*, 2016; Sherry *et al.*, 2017; Franzmann *et al.*, 2018; Staller *et al.*, 2018; Wang *et al.*, 2018; Beveridge *et al.*, 2019). Analysis of changes to specific sequence parameters on sequence-function/sequence-phenotype relationships have revealed the fact that no single compositional parameter can serve as an adequate design feature that connects IDP/IDR sequences to their functions (Das *et al.*, 2016; Sherry *et al.*, 2017; Staller *et al.*, 2018). What we require are quantitative measures that account for the totality of ensemble features encoded by IDP sequences (Lyle *et al.*, 2013).

**Fig. 1:**
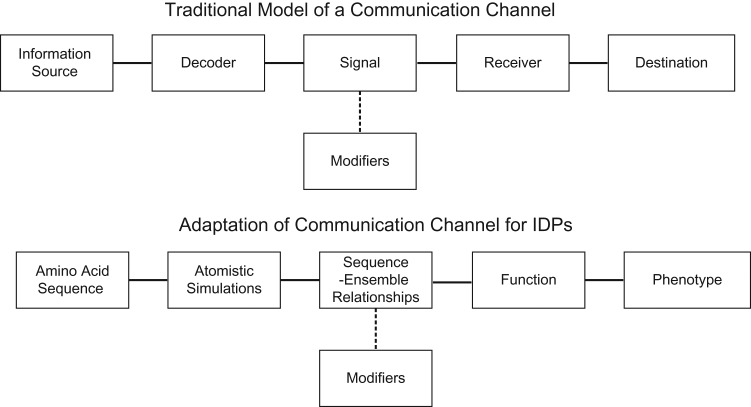
Adaptation of a communication channel to describe protein design, focusing on IDP design. In a traditional communication channel, the information source produces the message, which is then decoded and converted into a signal for transmission over the channel by the decoder. The actual signal that is transmitted is a convolution of the intrinsic signal and extrinsic modifications introduced in the form encryption, noise, or ancillary signals. The transmission is processed by a receiver and relayed to its intended destination. We propose that the model of a communication channel can be adapted to describe proteins, such that the amino acid sequence (information source) encodes protein function (receiver) and resulting cellular phenotype (destination). IDPs exhibit conformational heterogeneity. Therefore, analysis of all-atom simulations that considers the entire ensemble of conformations needs to be used to decode the information contained in the IDP sequence.

An analogy to the design of communication channels (Fig. 1) helps in making our point about the importance sequence-ensemble relationships for IDPs/IDRs (Csizmok *et al.*, 2016). In a traditional communication channel (Shannon, 1948), the information source produces the message, which is then decoded, and converted into a signal for transmission over the channel. The actual transmission is a convolution of the intrinsic signal and extrinsic modifications introduced in the form of encryption, noise, or ancillary signals. The transmission is processed by a receiver and relayed to its intended destination. In our conceptualization of the analogy to communication channels the information source is the protein sequence (Fig. 1). The key decoding unit that facilitates protein design and formalizes analogies between communication channels and sequence-structure-function channels are the sequence-encoded and decodable sequence-structure relationships. For IOPs, these can be gleaned by combining MSAs (Pirovano and Heringa, 2008) and structural comparisons (Marks *et al.*, 2011; Reynolds *et al.*, 2013; Hopf *et al.*, 2014). The situation is quite different for IDPs/IDRs because no singular structure provides a suitable representation or abstraction for the types of conformations that these sequences can adopt. Efforts over the past decade have uncovered a series of rules and heuristics that connect the sequences of IDPs/IDRs to conformational ensembles that they adopt (Mittag and Forman-Kay, 2007; Marsh and Forman-Kay, 2010; Kragelj *et al.*, 2013; Brucale *et al.*, 2014; Jensen and Blackledge, 2014; Liu *et al.*, 2014; Das, Ruff and Pappu, 2015; Csizmok *et al.*, 2016; Gibbs *et al.*, 2017). SERs should serve as quantitative proxies for sequence-structure relationships and pave the way to understanding and modulating how the information encoded in IDP/IDR sequences contributes to protein function and cellular phenotypes.

How are SERs quantified? Recent advances have enabled all atom simulations with sufficient throughput for a variety of IDPs/IDRs (Vitalis *et al.*, 2007; Turjanski *et al.*, 2008; Mao *et al.*, 2010; Vitalis and Caflisch, 2010, 2012; Das *et al.*, 2012, 2015; De Sancho and Best, 2012; Ganguly *et al.*, 2012; Radhakrishnan *et al.*, 2012; Best *et al.*, 2014; Wuttke *et al.*, 2014; Metskas and Rhoades, 2015; Piana *et al.*, 2015; Ruff *et al.*, 2015, 2018; Zerze *et al.*, 2015, 2019; Gibbs and Showalter, 2016; Fuertes *et al.*, 2017; Harmon *et al.*, 2017; Warner *et al.*, 2017; Dignon *et al.*, 2018; Mittal *et al.*, 2018; Newcombe *et al.*, 2018; Robustelli *et al.*, 2018; Zheng *et al.*, 2019). The use of implicit solvation models combined with advances in Monte Carlo sampling enables the efficiency required for being able to simulate large number of sequences derived from similar functional families (Vitalis and Pappu, 2009a, b, 2014; Radhakrishnan *et al.*, 2012; Das *et al.*, 2015; Mittal *et al.*, 2015; Ruff *et al.*, 2019). Comparisons to experiments suggest that the conformational ensembles that result from the use of efficient simulations based on implicit solvation models have the accuracy that should enable the construction of quantitative SERs (Das *et al.*, 2012; Fuertes *et al.*, 2017; Gibbs *et al.*, 2017; Warner *et al.*, 2017; Munshi *et al.*, 2018; Newcombe *et al.*, 2018). Further, these simulations can be combined with genetic algorithms to design IDPs/IDRs that fit specified criteria for SERs (Harmon *et al.*, 2016). What we require is a formal set of measures to quantify SERs for IDPs/IDRs. This, we propose, will allow us to uncover the design principles that connect information encoded in IDPs/IDRs to their functions and the cellular phenotypes they influence.

Given our analogy between protein design and the design of communication channels, we use methods from information theory to develop measures quantify SERs. We show that these measures enable large-scale comparisons of SERs across designed and naturally occurring sequence families. These measures reveal the inadequacies of using compositional parameters as the only parameters to be modulated for tuning sequence-encoded information in IDPs/IDRs. We find that sequences with similar compositional biases can have different SERs. We also find that highly dissimilar sequences can have similar SERs leading to disparate sequences being part of similar functional families across orthologs. This finding highlights one of the reasons for the large sequence dissimilarities that have been observed for IDPs/IDRs that belong to similar functional families across orthologs.

## Methods

### Simulations of sequences of FtsZ-CTTs

All-atom Monte Carlo simulations were performed using the ABSINTH implicit solvent model and forcefield paradigm as made available in version 2.0 CAMPARI simulation package (http://campari.sourceforge.net) (Vitalis and Pappu, 2009a, b; Radhakrishnan *et al.*, 2012). Simulations were based on the abs_3.2_opls.prm parameter set in conjunction with optimized parameters for neutralizing and excess Na^+^ and Cl^–^ ions (Mao and Pappu, 2012). Simulations were performed using a spherical droplet with a diameter of 285 Å with explicit ions to mimic a concentration of 10 mM NaCl. Temperature replica exchange Monte Carlo (T-REMC) (Sugita and Okamoto, 1999) was utilized to enhance conformational sampling. The temperature schedule ranged from 280 K to 400 K. Ensembles corresponding to a temperature of 310 K were used in the analysis reported in this work. Three independent sets of T-REX simulations were performed for each CTT sequence. In all, the ensembles for each CTT sequence were extracted from simulations, where each simulation deploys 4.6 × 10^7^ Monte Carlo steps. In each simulation, the first 10^6^ steps were discarded as equilibration. Simulation results were analyzed using the MDTraj and CTraj routines that are available at http://pappulab.wustl.edu/CTraj.html. The results for the RAM regions of the WT and designed variants of NICD were those of Sherry *et al.* and also use the ABSINTH model and were obtained using the CAMPARI engine.

## Results

**Fig. 2:**
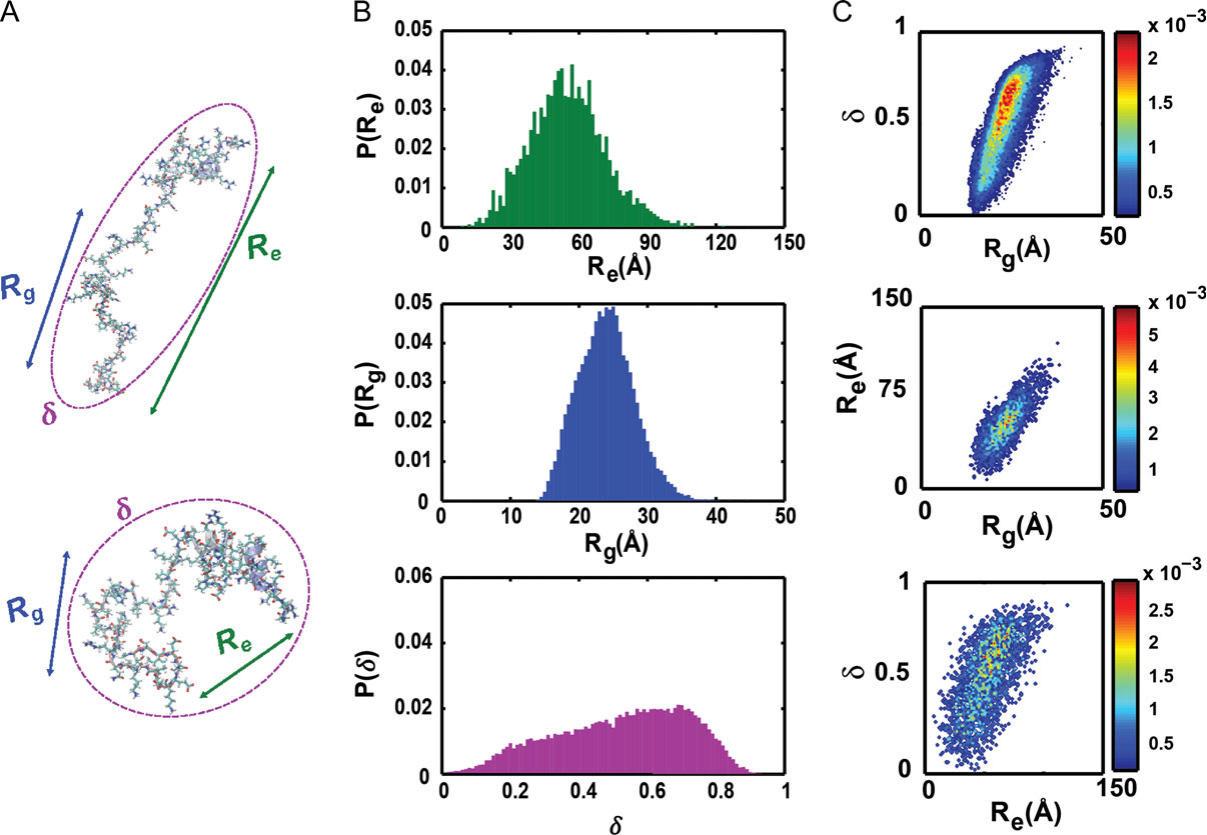
Illustration of conformational features of IDPs/IDRs extracted from all-atom simulations. (**A**) Two distinct conformations are shown from the disordered C-terminal tail of *B. subtilis* FtsZ. Each conformation within the ensemble has a set of properties. Here, we focus on three distinct properties: *R*_*g*_, *R*_*e*_ and δ (see text). **(B)** One-parameter marginal distributions of *p*(R_g_), *p*(R_e_) and *p*(δ) for the conformational ensemble of the disordered C-terminal tail of *B. subtilis* FtsZ **(C)** Contour plots of the resulting two-parameter marginal distributions of *p*(*R*_g_,δ), *p*(*R*_g_,*R*_e_) and *p*(δ,*R*_e_) shown with distribution-specific color bars.

All atom simulations of disordered systems may be performed in one of two modes: IDRs tethered to ordered domains can be simulated in their full protein contexts; alternatively, IDRs can be treated as an autonomous units and sequence-intrinsic conformational preferences of IDRs are then extracted from simulations. The latter mode is the more pervasive approach, although recent developments in enhanced sampling (Mittal *et al.*, 2014) have enabled the simulations of IDRs tethered as disordered tails to ordered domains or as linkers between ordered domains. A typical simulation will yield an ensemble of conformations that can be analyzed using a series of global and local parameters. We focus here on global parameters that are central to polymeric descriptions of heterogeneous systems namely, radii of gyration (*R*_g_), asphericity (δ), and end-to-end distance (*R*_e_) – see Fig. 2**A**. Each of the three parameters can be gleaned from small angle x-ray scattering (SAXS) measurements (Steinhauser, 2005), although the reliability of the estimate will depend on the parameter itself.

*R*_g_ quantifies the average distance, for a given conformation, of all of the atoms with respect to its centroid; accordingly, it is a measure of the overall size, primarily in terms of the density of the polymer in question. Analysis of a SAXS profile in the Guinier regime enables direct estimates of 〈*R*_g_〉 values averaged over the thermodynamically relevant ensemble of the system. For a given conformation, δ quantifies the shape of the polymer and it is calculated using the eigenvalues of the gyration tensor (Steinhauser, 2005). Ensemble-averaged values of δ, denoted as 〈δ〉, can be extracted by inferring parameters for the average inertial ellipsoid that describes all of the SAXS data for a given system (Fuertes *et al.*, 2017). Values of 〈δ〉 can also be extracted from measurements of rotational diffusion, although care must be taken when connecting quantities derived from hydrodynamic measurements to parameters that are derived from the inertial ellipsoid. Similar concerns apply to conversions of hydrodynamic radii (〈*R*_h_〉) from translational diffusion measurements to estimates of 〈*R*_g_〉 (Mao *et al.*, 2010). Finally, ensemble averaged values of *R*_e_, which refer to the ensemble averaged distance between the ends of a chain, can be inferred from SAXS measurements, but are more readily obtained from single molecule Förster resonance transfer (smFRET) measurements. For homopolymers in the ideal solvent limit, 〈*R*_g_〉 and 〈*R*_g_〉 differ from one another by a multiplicative factor. However, away from the ideal solvent limit and specifically for heteropolymers such as IDPs/IDRs, the conformation-specific and ensemble averaged values of *R*_g_ and *R*_e_ can be decoupled from one another.

For a given sequence, the values of *R*_g_, δ and *R*_e_ are conformation-specific, and for a heterogeneous ensemble of conformations converged, statistically robust simulations will yield a distribution of values for these parameters. Accordingly, to first-order, a complete description of conformational ensembles in terms of global features can be cast as a three-parameter probability density function *viz*., *p*(*R*_g_,δ,*R*_e_). Features of this three-parameter distribution can be gleaned from three different marginal one-parameter distributions, *p*(*R*_g_), *p*(δ), and *p*(*R*_e_)—see Fig. 2**B**—or three different marginal two-parameter distributions, *p*(*R*_g_,δ), *p*(*R*_g_,*R*_e_) and *p*(δ,*R*_e_)—see Fig. 2**C**. For a given sequence, the one- and two-parameter marginal distributions shown in Fig. 2**B** and **C** provide a visual and quantitative description of conformational heterogeneity. We use these distributions to compute quantitative SERs as described next.

### The ensemble entropy matrix

**Fig. 3:**
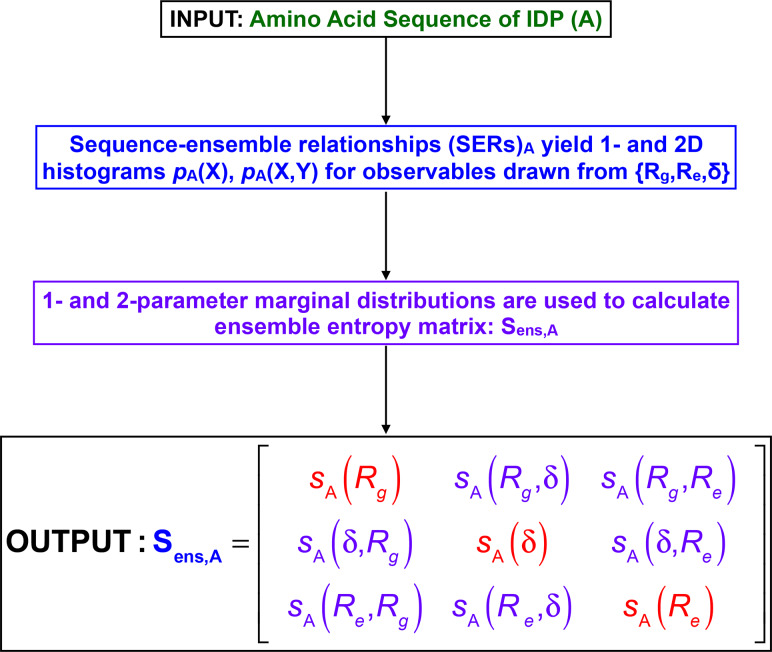
Summary of workflow used to generate the ensemble entropy matrix for a sequence of interest, designated as A.

**Fig. 4:**
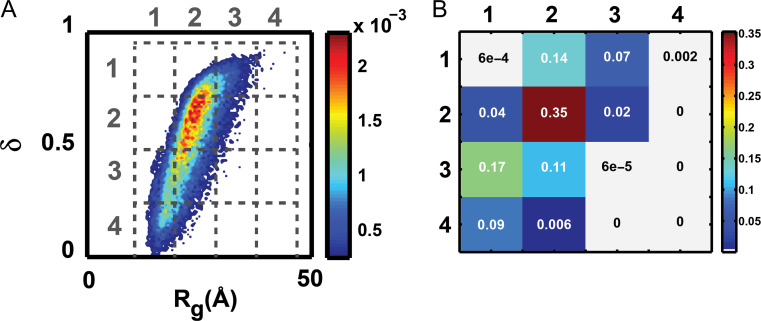
Example of a two-dimensional probability distribution of conformational properties used to quantify the information theoretic entropy (**A**) The two-parameter space is tiled into *n*×*n* grids. (**B**) Grid of probabilities derived from the 2-parameter distribution shown in (A).

Figure 3 summarizes the overall approach that we follow to arrive at a concise, quantitative, information theoretic description of the conformational ensemble for a given IDP sequence that is based on analysis of simulation results for one- and two-parameter marginal distributions. Consider the case of a two-parameter distribution *p*(*R*_g_,δ) shown in panel A of Fig. 4. The two-parameter space is tiled into a *n* × *n* grid and the integral of *p*(*R*_g_,δ) is computed for each of the grids, leading to a grid of probabilities as shown in panel B of Fig. 4 where, *n* = 4. In general, if (X,Y) are the parameters of interest, shown for (X,Y) ≡ (*R*_g_,δ) in panel B of Fig. 4, then the information theoretic entropy *s*(X,Y) using the grid of probabilities is computed as:

(1) $$s\left(X,Y\right)=-\overset{n}{\underset{i=1}{\sum }}\overset{n}{\underset{j=1}{\sum }}p\left(X_{i},Y_{j}\right)\ln\;p\left(X_{i},Y_{j}\right);$$

For a one-parameter distribution tiled into *n* bins, the corresponding information theoretic entropy is computed as:

(2) $$s\left(X\right)=-\overset{n}{\underset{i=1}{\sum }}p\left(X_{i}\right)\ln\;p\left(X_{i}\right);$$

The information theoretic entropies computed using a total of three one-parameter and three two-parameter marginal distributions become elements of symmetric square matrix that we refer to as the *ensemble entropy matrix*, denoted as **S**_ens_, and computed as:

(3) $$S_{\mathrm{ens}}=\left[\begin{array}{ccc} s\left(R_{g}\right) & s\left(R_{g},\delta \right) & s\left(R_{g},R_{e}\right)\\ s\left(\delta ,R_{g}\right) & s\left(\delta \right) & s\left(\delta ,R_{e}\right)\\ s\left(R_{e},R_{g}\right) & s\left(R{}_{e},\delta \right) & s\left(R_{e}\right) \end{array}\right];$$

**Fig. 5:**
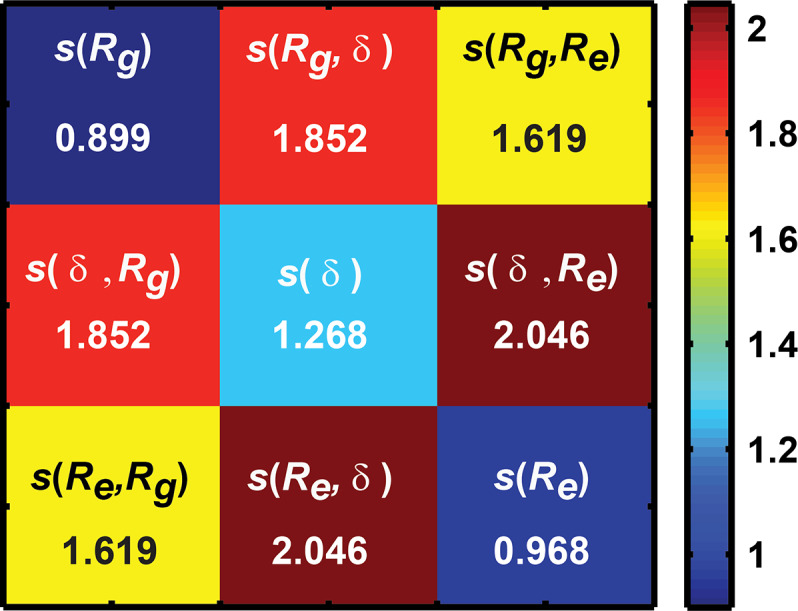
Using the one- and two-dimensional distributions to generate the ensemble entropy matrix: From the grid of probabilities for parameters X and Y (see Fig. 4) for *R*_g_ & δ, the information theoretic entropy *s*(X,Y) of the two-dimensional distribution is computed as $s\left(X,Y\right)=-\overset{n}{\underset{i=1}{\sum }}\overset{n}{\underset{j=1}{\sum }}p\left(X_{i},Y_{j}\right)\ln\;p\left(X_{i},Y_{j}\right)$. These values are shown as off diagonal elements of the ensemble entropy matrix (**S**_ens_). Entropies extracted from the one-parameter distributions are shown along the diagonal and are computed as: $s\left(X\right)=-\overset{n}{\underset{i=1}{\sum }}p\left(X_{i}\right)\ln\;p\left(X_{i}\right)$.

The ensemble entropy matrix provides a concise description of the SERs for a specific sequence. For an idealized maximally heterogeneous system with a flat distribution, setting *n* = 4 equal sized intervals will lead to upper limits of 1.386 for the diagonal elements and 2.773 for the off-diagonal elements of **S**_ens_. Fig. 5 shows the ensemble entropy matrix that we compute from all atom simulations for an archetypal polyampholytic IDP *viz*., the 67-residue disordered C-terminal tail (CTT) from the bacterial cell division protein FtsZ of *B*. *subtilis*.

### Comparative assessments of SERs

The ensemble entropy matrix can be calculated using simulation results for a sequence of interest. If we have a family of sequences, then the simulated ensembles for each sequence can be reduced to a sequence-specific ensemble entropy matrix. For a pair of sequences A and B, we shall denote the corresponding ensemble entropy matrices as **S**_ens,A_ and **S**_ens,B_, respectively. For this pair of sequences, we define a difference ensemble entropy matrix as: **∆**_AB_**=** (**S**_ens,A_ – **S**_ens,B_). The dissimilarity between SERs for sequences A and B is calculated as the Frobenius norm of the difference ensemble entropy matrix according to:

(4) $$D_{\mathrm{AB}}={\left\|\Delta_{\mathrm{AB}}\right\|}_{F}=\sqrt{\overset{3}{\underset{i=1}{\sum }}\overset{3}{\underset{j=i}{\sum }}{\left(\Delta_{ij}^{\left(\mathrm{AB}\right)}\right)}^{2}};$$

In Equation (4), ${\left\|\Delta_{AB}\right\|}_{F}$ denotes the Frobenius norm of ∆_AB_ and $\Delta_{ij}^{\left(\mathrm{AB}\right)}$ are the elements of ∆_AB_. If the SERs, described quantitatively in terms of **S**_ens_ matrices, are essentially identical for a pair of sequences A and B, then the D_AB_ → 0; conversely, for a pair of sequences whose SERs are maximally dissimilar, D_AB_ = 5.369. In reality, the constraints imposed by chain connectivity and excluded volume considerations imply that the upper bound will be considerably lower than the theoretical maximum that is set by assuming an ensemble defined by flat one- and two-parameter marginal distributions. However, the theoretical lower and upper bounds provide a natural rubric for comparing SERs across pairs of sequences. This is first illustrated for a set of sequences of identical length and amino acid composition. We then follow this up by using the **S**_ens_ matrix derived dissimilarity measures to compare SERs for sequences derived from the same functional family across orthologs.

### Comparative assessments of SERs across a set of sequences of identical lengths and amino acid compositions

A significant majority of IDP sequences are classified as strong polyampholytes. In these systems, the FCR values are greater than 0.3 whereas the NCPR values are close to zero. The calculated and measured values for ensemble averaged radii of gyration (〈*R*_g_〉) of strong polyampholytic IDPs are governed by the mixing vs. segregation of oppositely charged residues within the linear sequence (Das and Pappu, 2013). This feature, referred to as charge patterning, can be quantified by a parameter known as κ or other variants of this parameter (Sawle and Ghosh, 2015). For a fixed amino acid composition, one can design a series of sequence variants characterized by κ values that range from 0 to 1, where 0 implies sequences where the oppositely charged residues are evenly distributed along the linear sequence and 1 implies that the oppositely charged residues are segregated into distinct blocks along the sequence.

Recent investigations have quantified the impact of changes to κ on the global dimensions of different IDPs and the functions controlled by these IDPs (Das *et al.*, 2016; Sherry *et al.*, 2017). One such example is of the Notch intracellular domain (NICD), which controls the transcription of Notch genes that contribute to the determination of cell fate in metazoans (Johnson *et al.*, 2010; Kopan, 2012). NICD has a bipartite RAM-ANK architecture comprising of an IDR in the form of a 120-residue RAM region that is fused to an Ankyrin (ANK) repeat domain. Sherry *et al.* (2017) recently designed a set of NICD variants that have identical ANK domains but differ in the charge patterning of their RAM regions. The amino acid compositions of the RAM regions and the sequence of the motif that binds to the transcription factor CSL were also identical across the designed variants. In all, thirteen distinct NICD variants were designed, and the κ values of the RAM regions of these sequences are shown in Table I. Sherry *et al.* found that the computed 〈*R*_g_〉 values and measured hydrodynamic radii of RAM variants show an inverse correlation with κ (Sherry *et al.*, 2017). The Pearson *r-*values quantifying the inverse correlations were 0.895 and 0.858, respectively. Interestingly, the average asphericity values showed considerably weaker inverse correlation with κ with a Pearson *r*-value of 0.55.

**Table I.** Summary of κ values and parameters extracted from all atom simulations for the RAM region extracted from the WT and designed NICD variants. Data are from the work of Sherry *et al.*

Variant	κ	<R_g_ > Å	<R_e_ > Å	<ð>
WT	0.32	26.93	42.00	0.33
PT1	0.16	29.80	58.77	0.35
PT2s	0.21	28.19	46.66	0.31
PT3s	0.22	27.55	52.53	0.32
PT4	0.22	32.43	64.24	0.42
PT5s	0.32	27.43	46.21	0.32
PT6	0.40	28.14	53.46	0.33
PT7s	0.40	25.48	40.43	0.30
PT8s	0.44	26.23	49.55	0.31
PT9	0.45	26.40	48.67	0.36
PT10	0.50	25.04	45.41	0.28
PT11	0.55	24.85	47.05	0.26
PT12	0.60	24.85	49.19	0.37
PT13	0.75	24.65	42.88	0.28

**Fig. 6:**
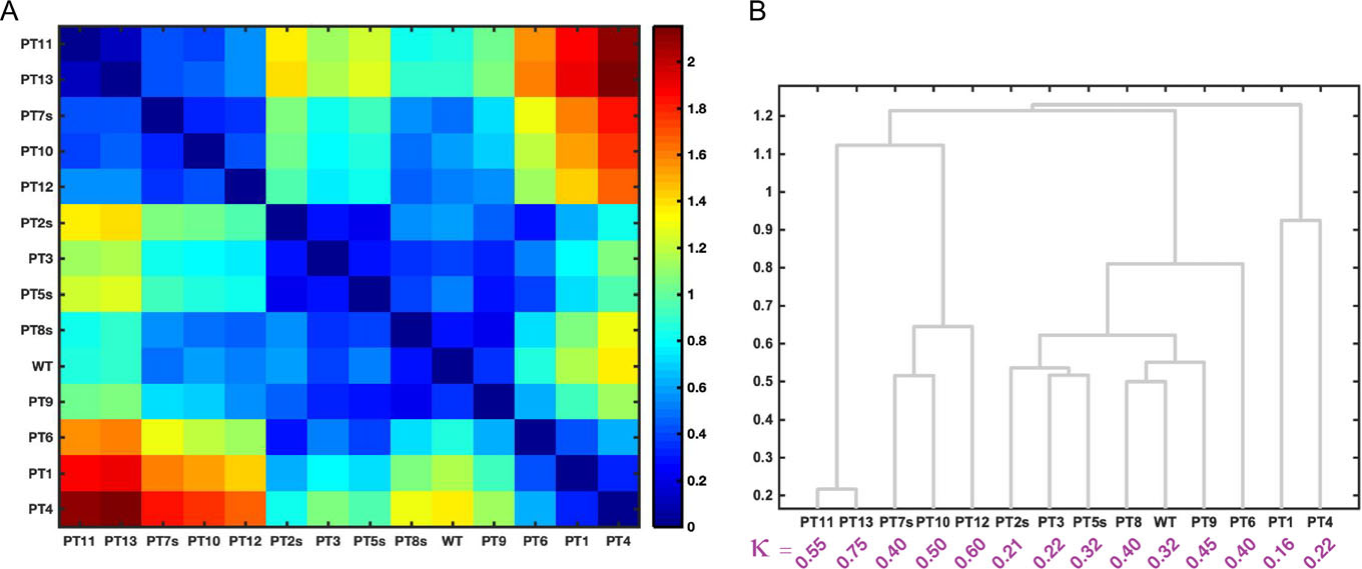
Comparative assessments of SERs for the RAM regions of NICD variants: (**A**) Checkerboard plot of the similarity between ensemble entropy matrices of the system of Notch-RAM variants. The dissimilarity between ensemble entropy matrices **S**_ens,A_ and **S**_ens,B_ is calculated as the Frobenius norm of the difference ensemble entropy matrix according to Equation (4). (**B**) Dendrogram of sequences grouped based on the similarities of their **S**_ens_ matrices.

We computed ensemble entropy matrices using results from all atom simulations (Sherry *et al.*, 2017) for each of the 13 RAM variants as well as the WT sequence. These matrices were used to compute pairwise dissimilarities (D_AB_) between SERs and the results are shown in the form of a checkerboard plot in panel A of Fig. 6. The RAM variants whose SERs are most similar to those of the WT sequence are PT8s (κ = 0.44), PT9 (κ = 0.45), PT3 (κ = 0.22), PT5s (κ = 0.32), and PT7s (κ = 0.40). The pairwise dissimilarity measures derived from ensemble entropy matrices were used to construct a dendrogram that arranges sequences in terms of extent of similarity of their SERs. This is shown in panel B of Fig. 6 for the RAM variants. This dendrogram provides a visual summary of the detailed information presented in the checkerboard plot of panel A. It highlights the fact that statistically robust conformational distributions generated from all atom simulations can be used to quantify similarities/dissimilarities between pairs of IDPs.

**Fig. 7:**
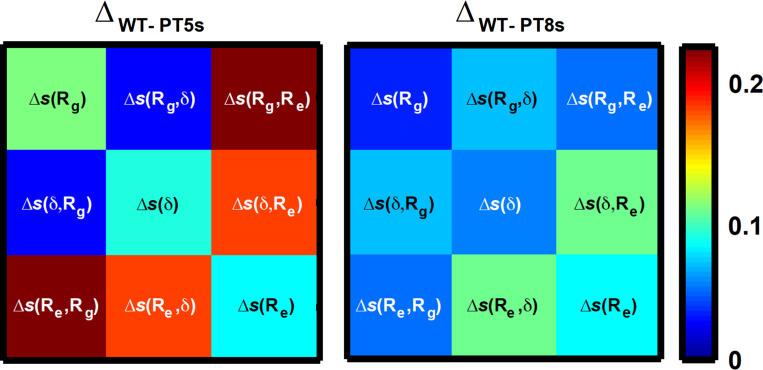
Comparison of the difference ensemble entropy matrices ∆_AB_ for the PT8s-WT and PT5s-WT pairs.

The results for the designed RAM variants help us illustrate the importance of using multi-parameter conformational distributions for quantifying and comparing SERs. To do so, we consider pairwise comparisons of the WT RAM with RAM variants of PT5s and PT8s, respectively. The dissimilarity measures D_AB_ are smallest for the PT8s-WT pair even though PT5s has a κ-value that is identical to WT RAM. Comparison of the difference ensemble entropy matrices ∆_AB_ for the PT8s-WT and PT5s-WT pairs, shown in Fig. 7, reveals the following: Despite having identical κ-values, identical ensemble-averaged *R*_g_ values, and very similar ensemble-averaged asphericity values, the two-parameter marginal distributions *p*(*R*_g_,*R*_e_) and *p*(δ,*R*_e_) are considerably different across the WT and PT5s sequences. This is underscored by the magnitudes of the differences between *s*(*R*_g_,*R*_e_) and *s*(δ,*R*_e_) for WT RAM vs. the RAM region from PT5s. These differences arise because of the sequence-specificity in size and shape fluctuations as well as sequence-specificity in the decoupling of *R*_g_ and *R*_e_ distributions. We also computed difference ensemble entropy matrices for the RAM regions of WT (κ = 0.32) and PT8s (κ = 0.44). Interestingly, while the ensemble-averaged R_g_, R_e_, and δ values of PT8s are more different from those of WT RAM when compared to PT5s vs. WT RAM (Table I), the overall dissimilarity measure D_AB_ is lowest for PT8s vis-à-vis the WT RAM.

The comparisons illustrated above highlight two crucial features of IDPs: similarities in the values of first moments of one-parameter marginal distributions such as such as 〈*R*_g_〉,〈*R*_e_〉, and 〈δ〉 can mask significant dissimilarities in the details of conformational ensembles. Secondly, dissimilarities in values for the first moments need not necessarily mean that the overall conformational ensembles have equivalent dissimilarities. Instead, conformational fluctuations can give rise to similar distributions, thereby offsetting differences in first moments. Conformational fluctuations are the defining hallmark of systems such as IDPs/IDRs and it is important to account for conformational distributions to account for quantitative similarities/dissimilarities between sequence specific ensembles. Our results emphasize the need for computing SERs using entire distributions, preferably computed in terms of multiple parameters, rather than over-interpreting changes observed from the scaling of first moments such as 〈*R*_g_〉 or 〈*R*_e_〉 with chain length, κ, or proxies for these parameters (Das and Pappu, 2013; Riback *et al.*, 2017; Firman and Ghosh, 2018).

### Quantitative SERs for IDRs derived from the same functional family across orthologs

**Fig. 8:**
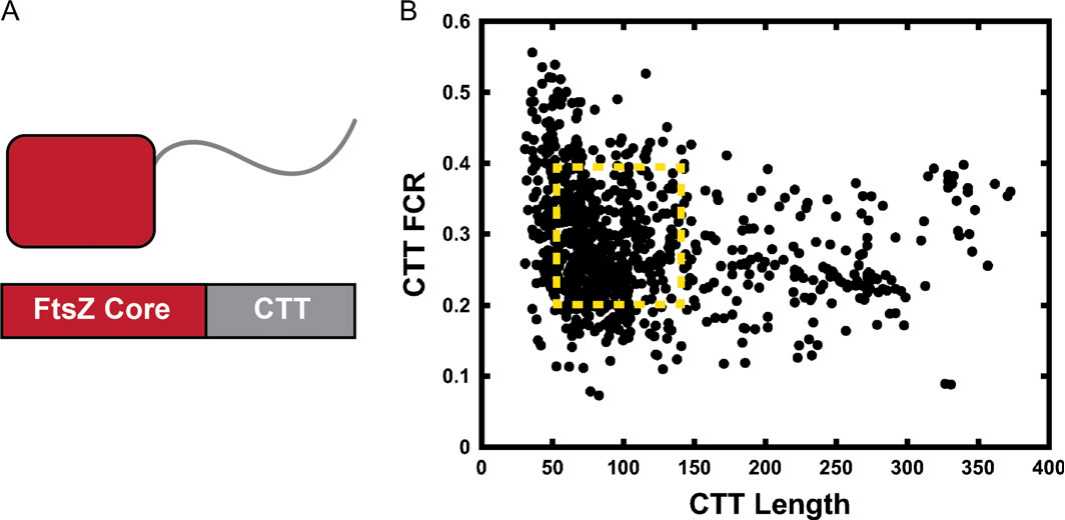
Details regarding FtsZ. (**A**) The protein comprises of an ordered GTPase core domain and a hyper-variable C-terminal tail (CTT). (**B**) Scatter plot of CTT sequence parameters summarized in terms of CTT-length and the Fraction of Charged Residues (FCR). To compare the conformational distributions of CTTs, we performed all-atom simulations for 58 distinct CTTs drawn from the yellow region (sequences are listed in **Table S1** of the **Supplementary Material**).

Covariation in sequence helps unmask cryptic sequence–structure relationships and this can be used to improve structure prediction, uncover the determinants of protein function, and enable novelties in protein design (Reynolds *et al.*, 2013; Raman *et al.*, 2016; Salinas and Ranganathan, 2018). Although covariation analysis has been adapted to predict the presence of specific structural motifs within IDPs/IDRs, this type of analysis requires large-scale MSAs and a high degree of sequence conservation as well. Most IDPs/IDRs are characterized by very poor sequence conservation. Examples of this include the RAM region of NICD (Bertagna *et al.*, 2008) that was discussed in the preceding section and the disordered C-terminal tail of the bacterial protein FtsZ (Buske *et al.*, 2015). Cell division in rod-shaped bacteria involves the polymerization and assembly of FtsZ into so-called Z-rings that form at the midsection of the dividing cell (den Blaauwen *et al.*, 2017; Wehrens *et al.*, 2018). FtsZ, which is a bacterial homolog of tubulin, is also a GTPase and it has a sequence architecture that is modular (Fig. 8**A**). The GTPase, referred to as the core, is an ordered domain that has a C-terminal tail (CTT) attached to it. The CTT is essential for Z-ring formation *in vivo* in bacteria where this has been studied (Buske and Levin, 2013).

A recent analysis quantified a variety of sequence features for CTT sequences derived from 1208 different FtsZ proteins across bacterial orthologs (Buske *et al.*, 2015). A summary of this analysis in Fig. 8**B** shows a scatter plot in a two-parameter space defined by CTT length and CTT FCR. This analysis suggests a confounding level of variation for the CTT sequences. The average CTT sequence is 60–70 residues long with an average FCR value of 0.3. However, there are significant deviations from the average values for CTT length and FCR. In fact, the distribution of points in Fig. 8 have an overall L-shape suggesting that longer CTTs tend to have lower FCR values, whereas shorter sequences have higher FCR values. A recent study showed that the relevant parameter for disordered linkers and tails is the so-called thermodynamic or *effective length* (Mittal *et al.*, 2018) and not the number of number of residues within an IDR sequence, which we refer to as the apparent length. Instead, the effective length is governed by the thermodynamically preferred end-to-end distance realized by the disordered linker or tail that is encoded by at least two parameters namely, the apparent length and the FCR, and is also influenced by extrinsic parameters such as solution conditions.

**Fig. 9:**
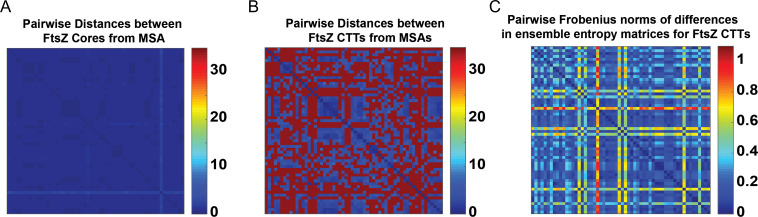
The conservation of the FtsZ core is in stark contrast to the hyper-variability of the FtsZ CTT sequences and resulting SERs. (**A**) Checkerboard plot of the pairwise distances extracted from a MSA shows minimal variation between pairs of cores. (**B**) Data from MSAs of CTT sequences exhibit a bimodality whereby the sequences are either very similar or very different from one another. The colorbar used to annotate panels (A) and (B) are the same. (**C**) Checkerboard plot of pairwise differences between ensemble entropy matrices for FTsZ CTTs.

Covariation in the values of apparent lengths and FCR suggests that there are likely to be preferred conformational distributions encoded by functional CTTs. To quantify and compare these conformational distributions, we performed all atom simulations for 58 distinct CTTs. The sequences of these CTTs span a range of length and FCR values and they are drawn from the bounded region within Fig. 8**B.** For our analysis, we first computed the distances between pairs of sequences for FtsZ cores that were derived from MSAs of the cores alone. As expected, the sequences of the core GTPase domains of FtsZ proteins show minimal variation across orthologs (Fig. 9**A)**. In contrast, a similar analysis, performed on the CTT sequences, we find that the pairwise sequence similarities have an apparent bimodality (Fig. 9**B**); pairs of CTT sequences are either highly similar or highly dissimilar. While the former is expected, the latter is surprising because the CTTs are essential for FtsZ function, and yet there appear to be a range of very different sequences that can contribute as functional CTTs.

We used simulation results for 58 different CTTs and computed pairwise dissimilarity measures (D_AB_) using the SERs that we obtained for each of the CTTs. The results are shown as a checkerboard plot in Fig. 9**C**. Since the IDR lengths differ for this analysis, we normalized the length-dependent parameters (*R*_g_ and *R*_e_) by the square root of the length prior to computing the one- and two-parameter marginal distributions that are required to construct the ensemble entropy matrices. The bimodality that is apparent in the comparison of CTT sequences is not manifest in the D_AB_ that are used to quantify similarities/dissimilarities in SERs.

**Fig. 10:**
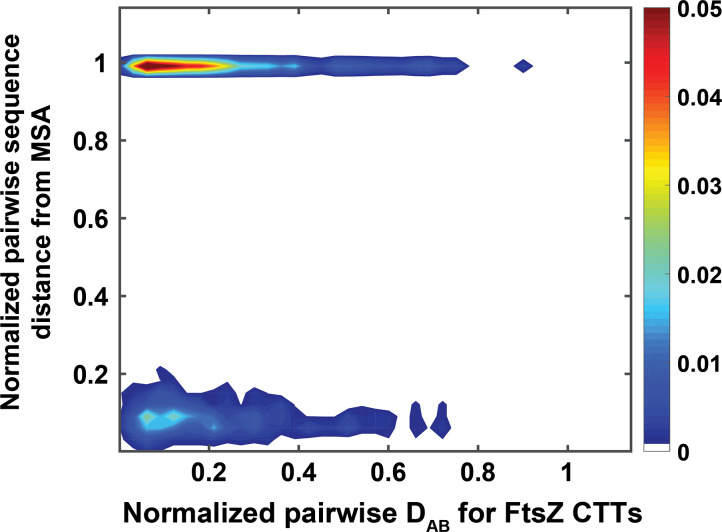
Histogram of SERs and sequence similarities. Two-dimensional histogram of the normalized pairwise CTT D_AB_ values and normalized pairwise distances between aligned CTT sequences. Both distributions are normalized by their maximum values.

Next, we computed the Frobenius norms of pairwise difference ensemble entropy matrices and normalized these values by the maximal norms. We also computed normalized pairwise distances between CTT sequences, where the normalization was performed using the maximal difference between sequences. Figure 10 shows a two-dimensional histogram of the two normalized values computed for all 58 FtsZ-CTT sequences. If the normalized Frobenius norms of pairwise difference ensemble entropy matrices *and* normalized pairwise sequence differences are less than 0.5, the implication is that similar sequences will have similar SERs. This region, which corresponds to the lower left corner of the two-dimensional histogram, encompasses 40% of the CTTs studied here. If normalized Frobenius norms of pairwise difference ensemble entropy matrices and normalized pairwise sequence distance differences are both above 0.5, then the differences in CTT sequences translate to differences in SERs—corresponds to 7% of CTTs studied here. Interestingly, 46% of the CTTs studied here fall into the top left region of the 2-dimensional histogram. In this region, the normalized Frobenius norms of pairwise difference ensemble entropy matrices are below 0.5, whereas the normalized differences between sequences exceed 0.5. These CTT sequences have very similar SERs despite having very different sequences. Overall, the results highlight the value of analyzing SERs across sequences derived from orthologs. The overall implication is that for over 86% of the FtsZ CTTs the SERs, quantified in terms of multi-parameter conformational distributions, are largely preserved even though in 46% of these sequences the pairwise sequence comparisons show considerable divergence.

## Conclusions

IDPs/IDRs feature prominently in eukaryotic proteomes (Liu *et al.*, 2006). As semantic descriptions for systems exhibiting different degrees of conformational heterogeneity were being developed, terms such as intrinsically unstructured proteins were introduced (Wright and Dyson, 1999) and even used in the literature (Harauz *et al.*, 2004; Takahashi *et al.*, 2009; Huhn *et al.*, 2014; Bencivenga *et al.*, 2017). This phraseology can erroneously be taken to imply that IDPs are unstructured. However, being disordered and being unstructured are not the same (Smyth *et al.*, 2001) and this has become clear with intense efforts yielding formal and heuristic descriptions of sequence–ensemble relationships for IDPs/IDRs. Indeed, these sequences come in distinct flavors (Hofmann *et al.*, 2012; Das *et al.*, 2015; Holehouse *et al.*, 2017); and the types of ensembles that are accessible to IDPs/IDRs are governed by the information encoded within their sequence and influenced by a variety of extrinsic factors including solution conditions, concentrations of ligands and epigenetic modifications. Importantly, disorder refers to measures that quantify the multi-parameter, sequence-specific conformational distributions that reflect the interplay of chain-solvent and intra-chain interactions (Lyle *et al.*, 2013). In this context, it is worth noting that numerous bioinformatics servers predict disordered regions within protein sequences. These predictors are valuable because they identify, with reasonable accuracy, the regions that are likely to be IDPs/IDRs as opposed IOPs or intrinsically ordered domains. These types of disorder predictions serve as the starting point for quantitative studies of SERs. Having identified a disordered region, one can perform suitable all atom simulations of the requisite throughput to generate thermodynamically accurate and statistically robust descriptions of conformational ensembles. Unlike an IOP that can often be described in terms of one or a small set of structures, with atomic coordinates in models for the structures, one needs statistical descriptions of SERs. Here, we have introduced a three-parameter distribution function that we decompose into three one-parameter and three two-parameter marginal distributions to then construct an ensemble entropy matrix (**S**_ens_) for a given IDP/IDR sequence. These **S**_ens_ matrices serve to synthesize the SERs by quantifying the information contained in distribution functions. Construction of pairwise difference ensemble entropy matrices and the calculation of Frobenius norms of these difference matrices allow us to compare sequences to one another through the lens of their SERs.

Our results reveal interesting insights regarding SERs of IDPs/IDRs. Analysis of the RAM regions of NICD variants show that while a single sequence encoded parameter such as κ is useful for generating novel variants, it does not fully describe SERs. Interestingly, considerable attention has focused on the calculation/measurement of first moments of conformational distributions such as ensemble-averaged values for *R*_g_, *R*_e_ and asphericity. These quantities show coherent variations with parameters such as chain length and sequence patterning for homopolymers and low complexity systems such as block copolymers. These simple systems are defined by the homogeneity of interactions on all length scales. However, most IDPs/IDRs are *bona fide* finite-sized heteropolymers wherein the sidechains are of different sizes, feature different functional groups, and encode very different balances between sidechain-solvent and sidechain–sidechain interactions. These chemical details cannot be readily captured using coarse-grained approximations for heteropolymers (Ruff and Holehouse, 2017). Recent studies have highlighted the importance of chemical heterogeneity on decoupling size and shape fluctuations and also the fluctuations of *R*_g_ and *R*_e_ (Fuertes *et al.*, 2017; Song *et al.*, 2017; Peran *et al.*, 2019). This type of decoupling raises caution about inferring SERs purely from the scaling of ensemble-averaged values of *R*_g_, *R*_e_ or asphericity. Instead, a complete description of SERs requires measurements of sizes, shapes, and conformational distributions and/or dynamics (Jensen *et al.*, 2013, 2014). Alternatively, one can pursue the all atom simulations providing they are efficient enough to enable sufficient throughput while also ensuring that they are accurate.

Of course, one cannot be certain of the accuracy of forcefields that are used for simulations of IDPs/IDRs. Considerable efforts have gone into the refinement of forcefields that are interoperable with explicit representations of solvent molecules (Best *et al.*, 2014). We have largely focused on the development, testing, refinement, and deployment of the ABSINTH implicit solvation model and forcefield paradigm for the simulations of IDPs/IDRs. To date, this model, aided by a variety of enhanced sampling methods, has been used to make predictions of SERs for well over 3000 sequences of different lengths and sequence complexities. Recent efforts have also focused on simulations of IDRs as tails and linkers tethered to ordered domains (Mittal *et al.*, 2014, 2018). Where possible, these simulations have been used to make testable predictions for scrutiny by experiment or reanalyzed using reweighting approaches based on experimental data. A new version of ABSINTH, known as ABSINTH-C (Choi and Pappu, 2019), was developed to remedy inaccuracies in the descriptions of local conformational equilibria. At the level of the conformational distributions studied here, it appears that the two versions generate roughly equivalent results. We propose that it should be possible to deploy ABSINTH/ABSINTH-C based simulations in high throughput investigations of SERs for IDPs/IDRs and combine this with a modified version of a recently developed genetic algorithm for designing sequences with bespoke SERs (Harmon *et al.*, 2016).

**Fig. 11:**
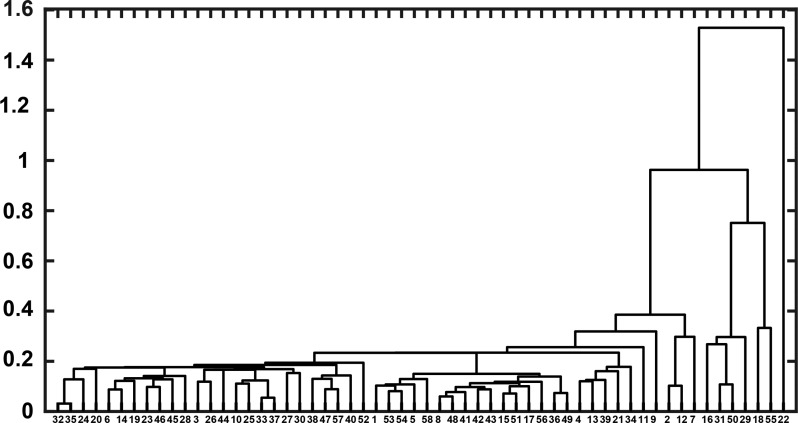
Dendrogram that arranges FtsZ CTT sequences based on similarities of SERs. The sequence IDs are derived from Table S1 shown in the Supplementary Material.

Our results for FtsZ CTTs underscore the importance of using SERs as a signal that can be modulated through sequence design. For example, one could conceive of a design strategy that generates a diverse library of CTTs that support bacterial cell division by ensuring the preservation of SERs. These designs can be guided by taxonomic inferences gleaned from a dendrogram that synthesizes all of the data in the matrix of all pairwise dissimilarity values. This dendrogram, shown in Fig. 11, is a similarity tree that groups CTT sequences based on the similarities of their SERs. We propose that to a first approximation, the CTTs with similar or identical SERs are likely to be functionally interoperable with one another. We anticipate that the use of SERs, fueled by advancements in computational efficiency and accuracy, will enable the emergence of new design paradigms that target the sequences of IDRs/IDPs for impacting cellular processes and circuits that are controlled by proteins with disordered regions.

## Supplementary Material

Table-S1_gzz014
